# Hemorrhagic Fever with Renal Syndrome Caused by 2 Lineages of Dobrava Hantavirus, Russia^1^

**DOI:** 10.3201/eid1404.071310

**Published:** 2008-04

**Authors:** Boris Klempa, Evgeniy A. Tkachenko, Tamara K. Dzagurova, Yulia V. Yunicheva, Vyacheslav G. Morozov, Natalia M. Okulova, Galina P. Slyusareva, Aleksey Smirnov, Detlev H. Kruger

**Affiliations:** * Charité School of Medicine, Berlin, Germany; † Slovak Academy of Sciences, Bratislava, Slovakia; ‡ Russian Academy of Medical Sciences, Moscow, Russia; 2 These authors contributed equally to this article.

**Keywords:** hantavirus, HFRS, Apodemus, research

## Abstract

Dobrava-Belgrade virus (DOBV) is a European hantavirus that causes hemorrhagic fever with renal syndrome (HFRS); case-fatality rates in Balkan countries are as high as 12%. To determine causative agents, we examined 126 cases of DOBV-associated HFRS in central and southern European Russia. In central Russia (Lipetsk, Voronezh, Orel regions), outbreaks were caused by a DOBV variant (DOBV-Aa) carried by *Apodemus agrarius*. In southern Russia (Sochi district), where HFRS is endemic, HFRS cases were caused by a new DOBV variant (DOBV-Ap), found in *A. ponticus,* a novel hantavirus natural host. Both viruses, DOBV-Aa/Lipetsk and DOBV-Ap/Sochi, were isolated through Vero E6 cells, genetically characterized, and used for serotyping of the HFRS patients’ serum. The clinical severity of HFRS caused by DOBV-Aa resembles that of HFRS caused by Puumala virus (mild to moderate); clinical severity of disease caused by DOBV-Ap infections is more often moderate to severe.

## Abstract 2

These lineages, including a new lineage associated with the novel rodent host *Apodemus ponticus*, are emerging.

In Russia, the zoonotic virus infection with the highest morbidity rate is hemorrhagic fever with renal syndrome (HFRS). This disease was first described in the 1930s as hemorrhagic nephroso-nephritis in far eastern Russia and Tula fever in European Russia (*1*). Since 1978, HFRS has been included in the official reporting system of the Russian Ministry of Public Health. Annually, 10,000–12,000 clinical cases of Puumala virus (PUUV) and Dobrava-Belgrade virus (DOBV) infection, mainly characterized by kidney failure, are reported from European Russia. Whereas PUUV infections predominantly occur in urban areas, 97% of DOBV-associated HFRS cases occur in rural environments (E.A. Tkachenko et al., unpub. data).

The hantaviruses, family *Bunyaviridae*, that cause HFRS are considered emerging viruses because of their increasing importance as human pathogens. Hantaviruses cause 2 human zoonoses: HFRS in Asia and Europe (caused by Hantaan virus [HTNV], Seoul virus [SEOV], PUUV, and DOBV) and hantavirus (cardio)pulmonary syndrome in the Americas (caused by Sin Nombre and Andes viruses) (*2*–*4*). Recently, 2 novel hantaviruses, with unknown pathogenic potential, were found in Africa (*5*,*6*).

Hantaviruses are transmitted by aerosolized excreta of their natural hosts, mainly rodents (family Muridae) but also shrews (family Soricidae). Particular hantavirus species are usually harbored each by a single or a few closely related rodent species. The virus genome contains 3 segments of negative-stranded RNA; the large (L) segment encodes viral RNA-dependent RNA polymerase, the medium (M) segment encodes glycoprotein precursor, and the small (S) segment encodes nucleocapsid protein (*4*).

DOBV seems to be the most life-threatening hantavirus in Europe; HFRS case-fatality rates are as high as 12% in Slovenia and Greece (*7*,*8*). DOBV was first isolated from a yellow-necked mouse (*Apodemus flavicollis*) captured in a natural focus of HFRS in Dobrava village, Slovenia (*9*). In the 1990s, an outbreak of HFRS in 2 regions of European Russia (Tula and Ryazan) was serologically confirmed to be caused by DOBV, but no clinical characterization of the patients was reported (*10*). In the Tula region, DOBV genetic material was detected in *A. agrarius* (striped field mouse), but not *A. flavicollis,* trapped in Kurkino village, a few kilometers from Tula city (*11*).

Molecular and serologic evidence indicate that in central Europe DOBV is harbored by *A. agrarius* and causes dozens of HFRS cases per year (*12*–*14*). This particular virus lineage is named DOBV-Aa, and a cell culture isolate of DOBV-Aa has been generated (*14*,*15*). The severity of HFRS caused by DOBV-Aa in central Europe is mild to moderate, less severe than its clinical course in Balkan countries associated with the DOBV-Af variant hosted by *A. flavicollis* (*14*,*16*,*17*).

Another genetic lineage of DOBV was found in the *A. agrarius* species on the Saaremaa island of Estonia, northeastern Europe; the Saaremaa virus has been established in cell culture (*18*). Later, these researchers postulated that Saaremaa virus should represent its own virus species separately from DOBV (*19*). Recently, 3 HFRS patients have been found by serologic approaches to have Saaremaa virus; however, no molecular (nucleotide sequence) identification of the involved virus strains has been reported (*20*).

Detailed phylogenetic analyses show that the strains from *A. flavicollis* form a separate evolutionary lineage (DOBV-Af) and that strains from *A. agrarius* show higher diversity. Strains from central Europe and European central Russia form the DOBV-Aa lineage, which is distinct from Saaremaa strains from northeastern Europe (*12*,*15*). DOBV ecology and evolution have recently become even more complex when DOBV was detected in an additional rodent host, *A. ponticus* (Caucasian wood mouse), captured in Sochi district, in the southern part of European Russia (*21*).

To determine causative agents, we examined 126 HFRS cases from 2 HFRS-endemic areas of European Russia. We isolated the viruses, genetically characterized them, and used them for serotyping.

## Material and Methods

### Patient Selection

During 2000–2006, blood samples from ≈600 patients around Sochi who had acute febrile illness with suspected hantavirus infection were tested for hantavirus antibody by an indirect immunofluorescence assay (IFA). Of these, 26 patients were found to be hantavirus antibody–positive. During the HFRS outbreak in the Lipetsk region in the winter of 2001–02, hantavirus infection of >100 patients was serologically confirmed by IFA.

### Rodent Trapping and Screening

Small mammals were trapped in the Sochi region during the 3 summer and autumn seasons of 2000–2002 and in the Lipetsk region during the winter of 2001–02. Lung tissues of the mammals were screened for the presence of hantavirus antigens by an antigen-capture ELISA as described (*22*).

### Virus Isolation

Suspensions (10%) of ELISA-antigen–positive lungs were added to Vero E6 cells as described (*23*). Then, with serum from HFRS patients, the cells were checked for hantavirus antigen by IFA. On the 32nd day of passage, positive cells were detected in a flask containing cells originating from an *A. agrarius* mouse from the Lipetsk region (Aa1854/Lipetsk-02 strain [Aa/Lipetsk]) and on the 70th day in another flask with cells from an *A. ponticus* mouse from Sochi (Ap1584/Sochi-01 [Ap/Sochi]).

### IFA

HFRS patient serum was screened for the presence of hantavirus antibody by IFA as described (*24*); slides with combined antigens from Vero E6 cells infected with PUUV, DOBV, HTNV, and SEOV were used as substrates. Slides with monovalent antigens of these viruses were used for serotyping hantavirus antibodies.

### Virus Titration and Focus-Reduction Neutralization Test

For confirmation and serotyping, all IFA-positive serum samples were tested by focus-reduction neutralization test (FRNT). The viral stocks, prepared from cell-culture supernatants of infected Vero E6 cells, were titrated with the chemiluminescence focus assay (*25*) or a protein A–peroxidase conjugate/DAB-NiCl_2_ (*26*). For FRNT, human convalescent serum samples were diluted serially in 2-fold steps, mixed with an equal volume of the respective virus containing 30–100 focus-forming units of this virus, incubated for 1 h at 37°C or overnight at 4°C–6°C, and then used to inoculate the cells. After 6–10 days of incubation, DOBV-N–specific rabbit antiserum or convalescent-phase human serum was used to detect the viral antigen as described above. A reduction in the number of foci of at least 80% was considered as the criterion for virus neutralization.

### Reverse Transcription–PCR, Cloning, and Sequencing

Hantavirus RNA was extracted from cell-culture supernatant by using the QIAamp Viral RNA Mini Kit (QIAGEN, Hilden, Germany). The standard QIAamp viral RNA mini spin protocol was performed. Amplification and sequencing of the entire S and M segments and partial L segment sequences were performed as described for the DOBV SK/Aa isolate (*15*).

### Sequence and Phylogenetic Analysis

Sequences were aligned by using ClustalW (*27*). The reliability of the alignment was checked by using DotPlot (*28*). The alignment was tested for phylogenetic information by likelihood-mapping analysis (*29*).

We calculated maximum-likelihood and neighbor-joining phylogenetic trees by using TREE-PUZZLE 5.2 (*30*) and PAUP* 4.0 Beta 10 software packages (*31*), respectively. The Tamura-Nei and Hasegawa-Kishino-Yano evolutionary models with and without gamma distribution of rate heterogeneity were used for the tree reconstructions. We used bootstrap analysis with 10,000 replicates to evaluate the statistical significance of the topology for the neighbor-joining trees. Similarity plots and bootscanning (*32*) were performed by using Stuart Ray’s SimPlot 3.2 with default parameters (*33*).

## Results

### HFRS Cases Associated with DOBV Infections

Altogether we characterized 126 patients with DOBV-associated HFRS (Table 1) from 2 geographically distant areas of European Russia. Of these, 108 were from the Lipetsk region (during 2001–02) and 18 were from the Sochi region (sporadic cases during 2000–2006).

**Table 1:** Characteristics of 126 patients with Dobrava-Belgrade–associated  hemorrhagic fever with renal syndrome, Russia*

Characteristic	Region, %	
	Sochi (2000–2006), n = 18	Lipetsk (2001–02), n = 108
Sex		
M	**94**	**66**
F	**6**	**34**
Age, y		
<16	12	7
17–59	88	82
>60	0	11

*Boldface indicates statistically significant differences between groups. Comparison of binomial population proportions analysis implemented in Statlets (NWP Associates, Inc.; www.mrs.umn.edu/~sungurea/statlets/statlets.htm) indicates that the null hypothesis that the 2 proportions are equal could be rejected at significance level of 5.0%.

In terms of clinical markers, a significantly higher proportion of the 18 patients from Sochi than from Lipetsk had abdominal pain, vision disturbance, nausea and vomiting, diarrhea, hyperemia of the face, hemorrhagic sclerae, liver enlargement, oliguria, and anuria; 1 patient died. On the other hand, hypertension developed in a significantly higher proportion of the 108 patients from Lipetsk; 1 patient died (Table 2).

**Table 2:** Clinical signs for 126 patients with Dobrava-Belgrade–associated  hemorrhagic fever with renal syndrome, Lipetsk (2001–02) and Sochi (2000–2006) regions, Russia*

Selected criteria	Region, %	
	Sochi, n = 18	Lipetsk, n = 108
Average duration of fever, d	7.1	5.4
Abdominal pain	**89**	**46**
Vision disturbance	**12**	**1**
Vomiting	**72**	**27**
Nausea	**89**	**44**
Diarrhea	**50**	**11**
Hyperemia of the face	**72**	**29**
Hemorrhagic sclerae	**50**	**2**
Hypertension	**6**	**34**
Liver enlargement	**83**	**23**
Oliguria (<500 mL)	**77**	**35**
Anuria (<200 mL)	**39**	**8**
Increased blood urea and creatinine	77	81
Death	**5.6**	0.9

*Boldface indicates statistically significant differences between groups. Comparison of binomial population proportions analysis implemented in Statlets (NWP Associates, Inc.; www.mrs.umn.edu/~sungurea/statlets/statlets.htm) indicates that the null hypothesis that the 2 proportions are equal could be rejected at significance level of 5.0%.

The clinical course of the disease was classified as mild, moderate, or severe, following the standard criteria used in the Russian Federation (*34*). According to these criteria, 55% of the cases in the Sochi region were classified as severe, 39% as moderate, and 6% as mild. In contrast, only 27% of the cases in the Lipetsk region were classified as severe, 54% as moderate, and 19% as mild (Table 3).

**Table 3:** Severity of clinical disease for 126 patients with Dobrava-Belgrade–associated hemorrhagic fever with renal syndrome, Russia*

Characteristic	Severity†		
	Mild	Moderate	Severe
Clinical sign or symptom			
Maximum temperature, °C	<38.0	38.0–39.5	>39.5
Headache	–/+	+/++	+++/++++
Vision disturbance	–	–/+	+/++
Low-back, abdominal pain	–/+	+/++	+++/++++
Hemorrhagic (petechial) skin rash	–	–/+	–/+/++
Oliguria (minimum mL/d)	>900	300–900	<200–300
Oliguria duration, d	6	9	11–13
Maximum blood urea, mmol/L	<8.3	8.3–19.0	>19.0
Maximum blood creatinine, μmol/L	<130	130–300	>300
Maximum leukocyte count, 10**^9^**/L	<8.0	8.0–14.0	>14.0
Clinical outcome by region			
Sochi (2000–2006)	6%	39%	**55%**
Lipetsk (2001–02)	19%	54%	**27%**

*Boldface indicates statistically significant differences between groups. Comparison of binomial population proportions analysis implemented in Statlets (NWP Associates, Inc.; www.mrs.umn.edu/~sungurea/statlets/statlets.htm) indicates that the null hypothesis that the 2 proportions are equal could be rejected at significance level of 5.0%. †According to Leshchinskaia et al. (*34*).

Association of all investigated HFRS cases with DOBV infection was established by IFA serotyping. Antibody titers against DOBV, HTNV, SEOV, and PUUV were determined. In all cases, antibody titers against PUUV were substantially lower. However, for most HFRS cases, IFA could not differentiate between DOBV, HTNV and SEOV; antibody specificities were finally characterized by FRNT.

### Rodent Trapping and Molecular Identification

During 2000–2002, an epizootiologic study was performed to identify and isolate the etiologic agents of the above-described HFRS cases (*21*,*26*). A total of ≈600 small animals (8 species) were trapped during 2000–2002 in the Sochi region and ≈300 animals (10 species) in the winter of 2001–2002 in the Lipetsk region. *A. ponticus* in the Sochi and *A. agrarius* in the Lipetsk region were the species that most frequently carried hantavirus antigen; 19.6% and 57.6% of animals, respectively, were positive according to ELISA.

**Figure 1:**
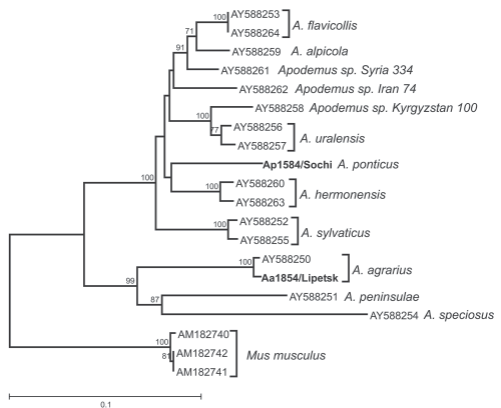
Phylogenetic analysis of D-loop sequences of the animal sources of the viruses Sochi/Ap and Lipetsk/Aa (in **boldface**): *Apodemus* *ponticus* from the Sochi region (Ap1584/Sochi; EU188455) and *A. agrarius* from Lipetsk region (Aa1854/Lipetsk; EU188456). Sequences of other *Apodemus* spp. were obtained from GenBank; accession numbers are indicated at the branch tips. The neighbor-joining tree was constructed by using the Tamura-Nei (TN93) evolutionary model. Values above the tree branches represent the bootstrap values calculated from 10,000 replicates. The scale bar indicates an evolutionary distance of 0.1 substitutions per position in the sequence.

To ensure correct classification of the reservoir hosts, tissue samples of the 2 animals that served as the sources of virus isolation were subjected to DNA extraction and sequence analysis. Nucleotide sequence of the mitochondrial DNA fragment containing the control region, D-loop, was determined for both animals and compared with *Apodemus* spp. D-loop sequences from GenBank. Neighbor-joining phylogenetic analysis demonstrated that the Aa1854/Lipetsk animal was identified correctly as *A. agrarius*. However, no *A. ponticus* D-loop nucleotide sequence was available in GenBank for comparison. Nevertheless, phylogenetic analysis of Ap1584/Sochi showed that the obtained D-loop sequence was distinct from all other analyzed sequences (Figure 1). This finding at least confirms that Ap1584/Sochi was not a misidentified member of *A. sylvaticus*, *A. flavicollis*, or another morphologically similar *Apodemus* species.

### Sequence Characterization of Virus Isolates

Complete S- and M-segment and partial L-segment nucleotide sequences of both isolates, Sochi/Ap and Lipetsk/Aa, were determined. The complete S segment of Sochi/Ap was found to be 1,649 nt long. It contained a single open reading frame (ORF; nt 36–1325) that encoded a putative nucleocapsid protein (N) of 429 amino acids. The complete S segment of Lipetsk/Aa was 24 nt longer (1,673 nt) due to a longer 3′ noncoding region. The Sochi/Ap M segment consisted of 3,616 nt that encoded a single ORF (nt 47–3448) of putative 1,133-aa glycoprotein precursor. The first putative start codon at positions 41–43, present in all other DOBV as well as HTNV M segment sequences, was missing, but the next one was located just 6 nt downstream in the same frame (as observed also in SEOV M-segment sequences). The M-segment sequence of Lipetsk/Aa was 3,643 nt long (ORF positions 41–3448; 1,135 aa); the difference in length is again the result of insertions/deletions in the 3′ noncoding region. In addition, a partial L-segment sequence of 541 nt (nt positions 109–649, according to the complete L-segment sequence of DOBV AP/Af; AJ410617) was determined for the Sochi/Ap and Lipetsk/Aa strains.

The sequence similarities between the 2 Russian DOBV isolates were rather low (Table 4). From the existing DOBV cell culture isolates, the Sochi/Ap strain shared the highest similarity with AP/Af19 isolate from Greece. Lipetsk/Aa virus was most similar to the SK/Aa strain. Between other available DOBV sequences, the Sochi/Ap virus S-segment sequence was highly similar to a partial sequence found in an HFRS patient from Krasnodar (P-s1223/Krasnodar-2000) as well as to the sequence Ap-1/Goryachiy Klyuch-2000 amplified from *A. ponticus.* (Krasnodar and Goryachiy Klyuch are places not far from Sochi.) As expected, the Lipetsk/Aa strain was most similar to Kurkino, another *A. agrarius*–associated strain from Russia (Table 4).

**Table 4:** Complete nucleotide and amino acid sequence identities of the Sochi/Ap and Lipetsk/Aa strains of Dobrava-Belgrade virus compared with currently available cell culture isolates and most related virus sequences of rodent and human origin*

Virus isolates	Sochi/Ap						Lipetsk/Aa				
	S segment			M segment			S segment			M segment	
	nt	aa		nt	aa		nt	aa		nt	aa
Sochi/Ap	–	–		–	–		86.6	96.7		79.7	91.3
Lipetsk/Aa	86.6	96.7		79.7	91.3		–	–		–	–
SK/Aa	84.8	97.4		78.6	90.4		89.9	98.8		87.2	97.0
Slo/Af	87.8	97.6		79.3	93.3		88.5	96.7		82.7	94.0
AP/Af19	87.6	97.9		79.6	93.3		88.2	97.4		82.5	94.1
Saa/160V	84.4	96.2		78.3	90.2		87.5	96.0		86.3	96.2
Kurkino/53Aa/98	86.6	96.7		NA	NA		98.8	99.5		NA	NA
Ap–1/Goryachiy Klyuch†	96.8	98.8		NA	NA		87.3	96.5		NA	NA
P–s1223/Krasnodar (patient)†	98.7	99.4		NA	NA		86.5	96.4		NA	NA

*NA, not available. †Values calculated from partial sequences available in GenBank (1,637 bp for Ap–1/Goryachiy Klyuch and 1,196 bp for P–s1223/Krasnodar).

### Phylogenetic Analysis

**Figure 2:**
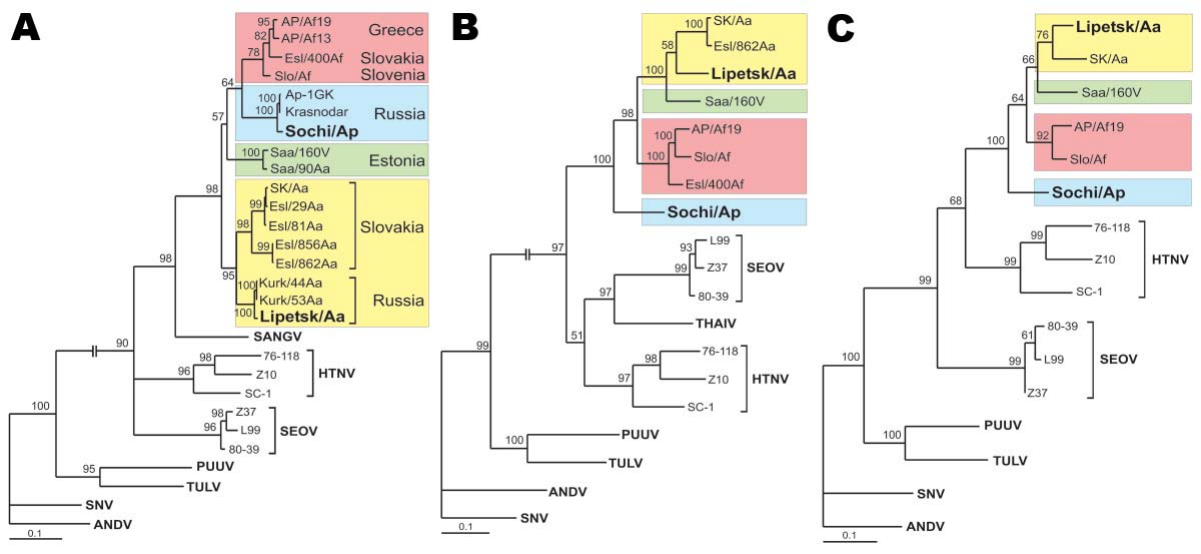
Maximum-likelihood phylogenetic trees of Dobrava-Belgrade virus (DOBV), showing the phylogenetic placement of novel Russian isolates Sochi/Ap and Lipetsk/Aa (in **boldface**) based on A) complete S-segment open reading frame (ORF) (nucleotide sequence positions 36–1325), B) complete M-segment ORF (nt positions 41–3445), and C) partial L-segment sequences (374 nt, positions 157–530). Different DOBV lineages are marked by colored boxes: yellow, DOBV-Aa; green, Saaremaa; red, DOBV-Af; blue, DOBV-Ap (Sochi virus). The Sochi/Ap and Lipetsk/Aa S-, M-, and L-segment sequences were deposited in GenBank under accession nos. EU188449–EU188454. The trees were computed with the TREE-PUZZLE package by using the Tamura Nei evolutionary model. The values at the tree branches are the PUZZLE support values. The scale bar indicates an evolutionary distance of 0.1 substitutions per position in the sequence. SANGV, Sangassou virus; HTNV, Hantaan virus; SEOV, Seoul virus; PUUV, Puumala virus; TULV, Tula virus; SNV, Sin Nombre virus, ANDV, Andes virus; THAIV, Thailand hantavirus.

Sequences of all 3 segments were analyzed by using maximum-likelihood and neighbor-joining phylogenetic methods with various evolutionary models. If not otherwise stated, all the trees for the particular dataset showed similar tree topology and statistical support, but only maximum-likelihood trees with Tamura-Nei evolutionary model are shown (Figure 2).

In the S-segment analysis, the Sochi/Ap sequence clustered with high statistical support with the patient-associated sequence from Krasnodar, Russia (AF442623), and the *A. ponticus*–associated sequence from the same region (AF442622) and formed a distinct lineage, which we named DOBV-Ap (Figure 2, **panel A**). Whereas DOBV-Ap and DOBV-Af share a common ancestor in the S-segment phylogenetic tree, in M and L segment analysis Sochi/Ap formed an outgroup from all other DOBV sequences and did not directly cluster with DOBV-Af sequences (Figure 2, **panels B**, **C**). Besides putative genetic reassortment processes, incomplete and unequal sequence datasets (fewer sequences for M- and L-segment datasets are available) could be the reason for these conflicting results. More sequence data are necessary to confirm these findings.

Lipetsk/Aa sequences unambiguously clustered within the DOBV-Aa lineage in the analyses of all 3 segments (Figure 2, **panels A–C**). As expected, it formed a well-supported monophyletic group with DOBV-Aa strains from Kurkino, Russia. In M and L segment analysis, for which the number of available sequences is rather limited, DOBV-Aa strains from Slovakia were most closely related, although the statistical support for this clustering was below the cutoff value of 70%.

We observed slight differences between maximum-likelihood and neighbor-joining trees in S segment analysis in which Saaremaa strains clustered with DOBV-Af and DOBV-Ap lineages in maximum-likelihood analyses but with DOBV-Aa in neighbor-joining analyses. However, in both instances, the statistical support was below the cutoff limit.

Moreover, we performed recombination analyses by using similarity plots and bootscanning. However, reliable results confirming homologous recombination events that affected Sochi/Ap or Lipetsk/Aa sequences could not be obtained (data not shown).

### Serotyping by Using the Novel Virus Isolates

The availability of the 2 novel DOBV strains as cell culture isolates enabled us to use them in chemiluminescence FRNT (c-FRNT) and characterize serum from selected patients also by neutralizing antibody titers. The DOBV prototype strain (Slo/Af; ref.1) and both novel isolates were used in c-FRNT to serotype all 18 patients from the Sochi region (27 serum samples; consecutive samples available for 8 patients). For 15 of these 18 patients, at least 1 serum sample showed the highest neutralizing antibody titer against the local Sochi virus. However, >4-fold differences in titers (considered to be significant) were found in serum samples of only 10 patients. For 8 patients, such significant difference was not found (5 patients with 2-fold difference, 3 with no difference). Table 5 shows examples of convalescent-phase serum samples representing these different groups.

**Table 5:** Results of typing of neutralizing antibodies in serum from patients with Dobrava-Belgrade virus–associated hemorrhagic fever with renal syndrome, Russia

Region	Sample no.	Time after onset of disease	FRNT titer* against						
			Sochi/Ap	Lipetsk/Aa	Slo/Af	SK/Aa	HTNV	SEOV	PUUV
Sochi (2000–2006)	1,312	104 d	2,560	80	160	ND	160	<80	<40
	3,692	30 d	1,280	160	160	ND	<80	<80	<40
	1,291	16 d	640	160	160	ND	40	40	<40
	4,714	1 y, 5 mo	>20,480	5,120	5,120	ND	320	320	<40
	1,310	50 d	2,560	640	640	ND	160	160	<40
	1,307	15 d	640	320	320	ND	160	160	<40
	4,716	5 y, 3 mo	5,120	1,280	2,560	ND	160	<80	<40
	4,715	1 y, 4 mo	5,120	2,560	5,120	ND	ND	ND	<40
Lipetsk (2001–2002)	4,338	6 mo	160	640	160	320	ND†	ND†	ND†
	3,894	21 d	40	640	40	160	ND†	ND†	ND†
	4,334	3 y, 6 mo	160	640	320	160	ND†	ND†	ND†
	4,344	6 mo	160	640	320	640	ND†	ND†	ND†
	3,958	3 mo	20	80	80	40	ND†	ND†	ND†
	4,329	3 y, 4 mo	640	640	2,560	640	ND†	ND†	ND†
Slovakia	B38	3 y, 9 mo	2,560	640	5,120	2,560	40	640	40
	B39	3 y, 9 mo	2,560	1,280	10,240	2,560	160	160	40

*FRNT, focus-reduction neutralization test. Reciprocal end-point titers are given as determined by chemiluminescence FRNT. ND, not determined. †Serum previously characterized as anti–Dobrava-Belgrade virus (DOBV) in a first FRNT investigation with Hantaan virus (HTNV), Seoul virus (SEOV), Puumala virus (PUUV), and DOBV–Slo/Af only.

From patients in the Lipetsk region, 6 serum samples were characterized by c-FRNT (Table 5). To verify whether some differences can also be found between 2 DOBV-Aa isolates, DOBV strain SK/Aa isolated in Slovakia, Central Europe (*15*), was included in the analysis. Four samples exhibited the highest titer against Lipetsk/Aa when compared with Slo/Af and Sochi/Ap, although in only 2 was the difference 4-fold. One sample reacted equally with Lipetsk/Aa and Slo/Af, and 1 showed even higher reactivity against the Slo/Af strain. When we directly compared Lipetsk/Aa and SK/Aa, equal proportions of serum showed equal titers and 2-fold or 4-fold higher titers against Lipetsk/Aa than against SK/Aa.

In addition, 2 DOBV convalescent-phase serum samples from Slovakia, previously serotyped as anti–DOBV-Af reactive (*15*), were analyzed to determine whether Sochi/Ap, Lipetsk/Aa, and Slo/Af could be distinguished by these samples. In both instances, Slo/Af virus was neutralized best, although in only 1 case was the difference in neutralizing antibody titer 4-fold (Table 5).

## Discussion

DOBV circulation was found in the Sochi region, southern part of Russia. We demonstrated that a new DOBV lineage (DOBV-Ap), associated with *A. ponticus* as a novel natural hantavirus host, was a causative agent of the human infection. Second, from an outbreak occurring in the Lipetsk region, central European Russia, >100 HRFS patients were characterized. This outbreak was found to be caused by DOBV-Aa infections. Both viruses, DOBV-Ap/Sochi and DOBV-Aa/Lipetsk, were isolated through Vero E6 cells, genetically characterized, and used for HFRS patient serotyping.

After the recent detection of DOBV RNA in several *A. ponticus* animals (*21*), isolation of viable virus can be taken as additional evidence that this rodent species represents a novel natural hantavirus host. Sequence and phylogenetic analysis showed that the strains from *A. ponticus* form a distinct lineage, which we propose to call DOBV-Ap. Moreover, clustering of the sequence previously found in a specimen from a patient with severe HFRS in Krasnodar near Sochi (*35*) represents final molecular evidence that DOBV-Ap causes HFRS in this region. The Sochi region is not yet considered to be a DOBV-associated HFRS-epidemic or -endemic region. Our findings therefore have some public health importance because this region is intensively used for recreation. DOBV-associated HFRS should therefore be considered for travelers returning from this region.

The Lipetsk area in Central European Russia is known for the DOBV outbreaks that occurred during 1991–1992 (130 registered cases) and 2001–2002 (167 registered cases, this study). During the winter of 2006–2007, this area faced a new large outbreak, which had ≈600 registered HFRS cases (authors’ unpub. data).

Serotyping of neutralizing antibodies confirmed as reasonable the assumptions that the HFRS cases in the Sochi region were caused by DOBV-Ap and that the Lipetsk outbreak was caused by DOBV-Aa strains. In this respect, the differences in clinical courses of infection for the Sochi and Lipetsk patients could be then also assigned as differences in the virulence of DOBV-Ap and DOBV-Aa lineages, respectively. Overall, the clinical course of DOBV-Aa infections in Lipetsk resembles that of PUUV infections observed in Russia (authors’ unpub. data), and the DOBV-Ap infections seem more often to be moderate to severe.

However, these differences should not be overestimated. Cases in the Lipetsk region occurred in an outbreak situation in a region where HFRS is a well-known disease. It is therefore possible that physicians and local authorities were much more aware of hantavirus infections and, therefore, also recognized those infections with mild clinical courses. In contrast, the sporadic cases in the Sochi region might be recognized only if the clinical course was severe. Differences in physician awareness in the 2 regions may result in a bias giving the impression that DOBV-Ap infections have a higher clinical severity. Alternatively, the higher virulence of DOBV-Ap might correspond with its close genetic relatedness with the DOBV-Af lineage, which causes rather severe disease in southeastern Europe. At the current stage of knowledge, the order of virulence of DOBV-like viruses in humans seems to be as follows: Saaremaa <DOBV-Aa <DOBV-Ap <DOBV-Af.

**Figure 3:**
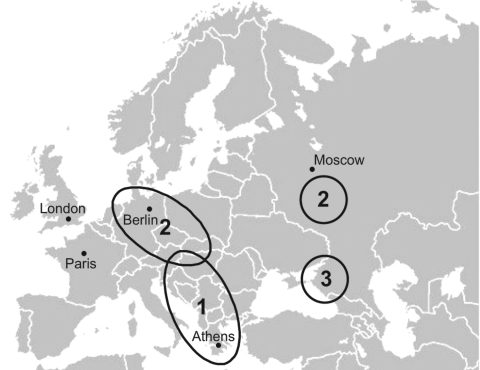
Map of Europe showing cases, identified by serologic as well as molecular methods, of hemorrhagic fever with renal syndrome caused by infection with the Dobrava-Belgrade virus (DOBV) variants: 1, DOBV-Af; 2, DOBV-Aa ; and 3, DOBV-Ap.

Figure 3 shows the regions in Europe where DOBV was demonstrated by serologic as well as molecular analyses to be the causative agent of well-characterized HFRS cases. Region 1 comprises the Balkan area in southeastern Europe where the classic DOBV (our DOBV-Af) was found in *A. flavicollis* animals as well as in human patients (*7*,*8*). Region 2 encompasses northeastern Germany and other regions of central Europe (*13*,*15*) as well as the central part of European Russia (this study) where the DOBV-Aa variant from *A. agrarius* causes mainly mild to moderate HFRS but also severe, life-threatening disease (*17*). The Sochi region, with its novel animal reservoir of DOBV, *A. ponticus*, and the DOBV-Ap–associated HFRS cases is marked as region 3. In all these areas, PUUV also circulates as an HFRS agent.

In addition to the public health aspect, our findings add another stone into the very complex mosaic of DOBV ecology and evolution. The Sochi/Ap virus is the first cell culture isolate of novel evolutionary lineage DOBV-Ap. *A. ponticus* is the third rodent species that should be considered a natural host of DOBV. Lipetsk/Aa is a new DOBV strain isolated on cell culture from *A. agrarius* (after Saaremaa virus from Estonia and SK/Aa from Slovakia) and the first originating from Russia and the first isolated in an outbreak region.

Rather unusual for hantaviruses, DOBV has already been found in 3 *Apodemus* species. Nevertheless, other hantaviruses are harbored by >1 (related) host species, e.g., Tula virus has been found in *Microtus arvalis, M. rossiaemeridionalis,* and *M. agrestis* (*36*–*38*) and SEOV in *Rattus rattus* and *R. norvegicus* (*39*). Although the DOBV strains from different *Apodemus* hosts share high amino acid sequence similarity, they can be distinguished in phylogenetic analyses as distinct lineages and seem to possess different virulence in humans as well as in an animal model (*40*). The novel DOBV-Ap lineage associated with *A. ponticus* emerging in an area south of European Russia confirms the reputation of DOBV being the most virulent of the European hantaviruses.
